# The application of personalized medicine in neurooncology

**DOI:** 10.1186/1878-5085-5-S1-A31

**Published:** 2014-02-11

**Authors:** Jiri Polivka Jr, Jiri Polivka, Vladimir Rohan, Ondrej Topolcan

**Affiliations:** 1 Department of Histology and Embryology, Faculty of Medicine in Pilsen, Charles University in Prague, Czech Republic; 2 Biomedical Centre, Faculty of Medicine in Pilsen, Charles University in Prague, Czech Republic; 3 Department of Neurology, Faculty of Medicine in Pilsen, Charles University in Prague and University Hospital Pilsen, Czech Republic; 4 Department of Nuclear Medicine, Immunoanalytic Laboratory, University Hospital Pilsen, Czech Republic

## 

Primary brain tumors form an important public health problem. There were more than 66,000 cases diagnosed in the US in 2012 with 13,700 deaths. The most malignant primary brain tumor in adults - Glioblastoma multiforme (GBM) - is extremely aggressive and invasive cancer, difficult to surgery with high resistance to standard radiotherapy and chemotherapy. The median survival of patient with GBM is 12.1 – 14.6 months and only 3-5% of patients survive longer than 3 years. There is an urgent need for further progress in neurooncology in the area of tumor early diagnosis, accurate characterization and more efficient treatment. The unprecedented progress of recent years in all omics disciplines such as genomics, transcriptomics, proteomics and others together with the improvements of bioinformatics technologies provides new opportunities in current brain cancer research. The human genome was fully sequenced and the improvements of sequencing methods have lately permitted genome-wide association studies in human cancers including primary brain tumors. The new milestones of oncogenesis - abnormalities in signaling pathways, tumor microenvironment, pathological angiogenesis, cancer metabolism and others – were uncovered over the past decade. Currently some novel molecular biomarkers in neurooncology are emerging that could be used as a prognostic and predictive factor of the disease. For glioblastoma multiforme the most promising biomarker, promoter methylation status of the MGMT gene, helps to predict the tumor response to standard chemotherapy with temozolomide. For all astrocytic tumors, there is a strong prognostic effect of mutations in the fundamental metabolic enzymes - Isocitrate dehydrogenases 1 and 2. Also the epigenetic changes of cancer genome, glioma cytosine-guanine island methylator phenotype, show promise like the prognostic biomarker. For oligodendrogliomas, the most promising predictive biomarker in relation to the standard PCV chemotherapy treatment is the loss of 1p/19q due to an unbalanced chromosomal translocation. There are many other potential markers for primary brain tumors in research, such as the amount of CD 133 positive cancer „stem“ cells, small non-coding microRNA’s or tumors gene expression profiles. With the discovery of novel molecular prognostic and predictive markers for various brain tumors, the therapy goal is rapidly changing from treating a class of tumors to the individual therapy plan for each patient according to the molecular characterization of the actual tumor, the real philosophy of personalized medicine.

Supported by Ministry of Health, Czech Republic - conceptual development of research organization (Faculty Hospital Pilsen - FNPl, 00669806) and by the project ED2.1.00/03.0076 from European Regional Development Fund.

